# Integration of Transcriptomics and Metabolomics for Pepper (*Capsicum annuum* L.) in Response to Heat Stress

**DOI:** 10.3390/ijms20205042

**Published:** 2019-10-11

**Authors:** Jing Wang, Junheng Lv, Zhoubin Liu, Yuhua Liu, Jingshuang Song, Yanqing Ma, Lijun Ou, Xilu Zhang, Chengliang Liang, Fei Wang, Niran Juntawong, Chunhai Jiao, Wenchao Chen, Xuexiao Zou

**Affiliations:** 1 Longping Branch, Graduate School of Hunan University, Changsha 410125, China; wangjinghnu@hnu.edu.cn (J.W.); junhenglv@hnu.edu.cn (J.L.); hnliuzhoubin@163.com (Z.L.); liuyuhua@hnu.edu.cn (Y.L.); sjs122912@163.com (J.S.); 2 Hunan Vegetable Research Institute, Changsha 410125, China; yanqingmahn@163.com (Y.M.); ytuwangjing@163.com (X.Z.); chenwenchao2019@163.com (C.L.); 3 College of Horticulture and Landscape, Hunan Agricultural University, Changsha 410125, China; ou9572@126.com; 4 Hubei Academy of Agricultural Sciences, Wuhan 430064, China; raymond@163.com; 5 Department of Botany, Faculty of Science, Kasetsart University, Bangkok 10900, Thailand; niranjuntawong@yahoo.com

**Keywords:** transcriptome, metabolome, heat shock protein, heat shock transcription factors, glutathione

## Abstract

Heat stress (HS), caused by extremely high temperatures, is one of the most severe forms of abiotic stress in pepper. In the present study, we studied the transcriptome and metabolome of a heat-tolerant cultivar (17CL30) and a heat-sensitive cultivar (05S180) under HS. Briefly, we identified 5754 and 5756 differentially expressed genes (DEGs) in 17CL30 and 05S180, respectively. Moreover, we also identified 94 and 108 differentially accumulated metabolites (DAMs) in 17CL30 and 05S180, respectively. Interestingly, there were many common HS-responsive genes (approximately 30%) in both pepper cultivars, despite the expression patterns of these HS-responsive genes being different in both cultivars. Notably, the expression changes of the most common HS-responsive genes were typically much more significant in 17CL30, which might explain why 17CL30 was more heat tolerant. Similar results were also obtained from metabolome data, especially amino acids, organic acids, flavonoids, and sugars. The changes in numerous genes and metabolites emphasized the complex response mechanisms involved in HS in pepper. Collectively, our study suggested that the glutathione metabolic pathway played a critical role in pepper response to HS and the higher accumulation ability of related genes and metabolites might be one of the primary reasons contributing to the heat resistance.

## 1. Introduction

Heat stress (HS) induces irreversible damage to plants when the ambient temperature is continuously higher than the optimum temperature for plants [1]. Since global warming caused by greenhouse gas emissions is increasing in severity, HS is becoming one of most severe forms of plant abiotic stress that destroys homeostasis, limit plant growth, and development, and even cause death [2,3,4]. Therefore, it is vital to elucidate the mechanisms of plants in response to HS. Pepper (*Capsicum annuum* L.), which is native to the tropics in Central and South America, is the most widely cultivated condiment and vegetable crop in almost all parts of the world [5,6,7]. Many environmental factors, such as water, salinity, heavy metals, and temperature, have distinct impacts on the life cycle of pepper. However, temperature is an extremely important factor that affects the growth and development of pepper. The optimum temperature for the growth of pepper ranges from 20 to 30 °C. Pepper is subject to HS when the ambient temperature is above 32 °C [8]. HS affects pollen development and pollen viability, leading to pollen abortion and causing a sharp reduction in pepper production [9]. As a result, how pepper responds to HS has become a hot topic.

Along with the rapid development of sequencing technology, transcriptomic analysis is now becoming increasingly common and efficient. Many outstanding studies on transcriptome reprogramming under HS in plants have been reported [10,11,12]. Many HS-responsive genes have been identified, including heat shock proteins (*HSPs*) and heat shock transcription factors (*HSFs*). *HSPs*, including small HSPs (*sHSPs*), *HSP40*, *HSP60*, *HSP70*, *HSP90*, and *HSP100* [13,14], which have been identified as molecular chaperones to promote protein folding, are up-regulated during HS [14,15,16]. HSP70 synthesis is prominently increased under HS in maize, and it interacts with individual enzymes to enhance plant tolerance [17]. Moreover, the over-expression of *OsHSP26* enhances the tolerance against HS in tall fescue [18]. *HSFs* (*HSFA*, *HSFB,* and *HSFC*), which are activated when plants are subjected to heat shock or HS, activate HSPs by binding to the heat shock element [19,20]. *HSFA1* has been identified as a master regulator of the HS response in tomato [21]. In Arabidopsis, *HSFB1* and *HSFB2b* function as transcriptional repressors and repress the expressions of HS-induced *HSFs* and several *HSPs* [22]. *HSFC1b* responds to HS, and the over-expression of *FaHSFC1b* enhances the survival rate of Arabidopsis under HS [23]. In addition, several transcription factors (TFs), such as *WRKY* [24], *MYB* [25], and *NAC* [26], are also involved in HS response. 

Furthermore, the complex metabolic regulatory networks also play important roles in response to HS in plants [27]. It has been reported that HS leads to a reorganization of the metabolic state to ensure homeostasis [28,29]. For example, protein [30], free proline [31], glycinebetaine [32], soluble sugars [33], phenolic compounds [28,34], and lipids [35,36] are related to HS response. Meanwhile, these metabolites not only confer HS tolerance by reducing oxidation and maintaining osmotic balance but also participate in metabolite synthesis in plants in response to HS. Metabolomic analysis has become an efficient tool for studying the responses of plants to biotic and abiotic stresses [27,37,38,39].

Due to the great development of bioinformatic tools and resources, it is becoming increasingly common and efficient to analyze the complex response process under environmental stress by integrating multi-omics data [40]. For instance, metabolites and genes involved in response to sulfur deficiency have been identified by using the integration of metabolomic and transcriptomic data [41], and the content of rutin is enhanced by over-expressing *AtMYB12* to improve insect resistance in tobacco [42]. In addition, some candidate genes and metabolites in the adaptation of oat plants to phosphor deficiency have been identified by transcriptomic and metabolomic analyses [43]. 

At present, it is widely accepted that the molecular mechanism of HS response in plants is highly complicated, which depends on diversified signal transduction pathways, genes, and metabolites [14,29]. Therefore, it is feasible to perform research using an integrated transcriptomic and metabolomic method.

In the present study, pepper seedlings were exposed to a temperature of 40 °C in a light incubator to simulate the high-temperature environment in summer. We compared the transcriptome and metabolome between the heat-tolerant variety 17CL30 and heat-sensitive variety 05S180 in response to HS at the seedling stage (six-leaf stage). We aimed to elucidate gene-gene, metabolome-metabolome, and gene-metabolome regulatory networks involved in the HS response and reveal the underlying mechanisms related to HS in pepper. Collectively, our findings showed that genes and metabolites could improve the heat tolerance of pepper and provide a theoretical basis for cultivating high-quality and heat-resistant varieties.

## 2. Results

### 2.1. Phenotype and Physiology Responses of 17CL30 and 05S180 under HS 

**Figure 1:**
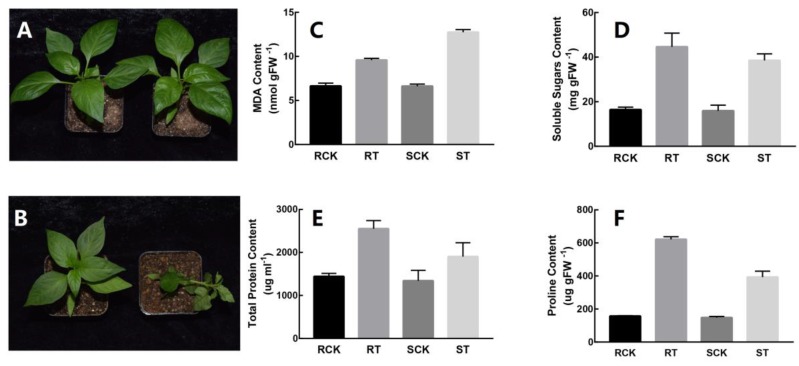
Phenotypic and physiological responses of 17CL30 (heat-tolerant cultivar) and 05S180 (heat-sensitive cultivar) under heat stress. RCK refers to the control group of the 17CL30, RT refers to the heat treatment group of 17CL30, SCK refers to the control group of 05S180, ST refers to the heat treatment group of 05S180. (**A**) Phenotype of heat-tolerant 17CL30 in control and heat-treated groups at 40 °C. (**B**) Heat-susceptible 05S180 in control and heat-treated groups at 40 °C. (**C**) Changes in malondialdehyde (MDA) content in the pepper leaves of both cultivars. **(D)** Changes in the content of total soluble sugars in the pepper leaves of both cultivars. (**E**) Changes in the content of total protein in the pepper leaves of both cultivars. (**F**) Changes in the content of proline in the pepper leaves of both cultivars.

The leaf tissues of pepper were used to study HS responses in the seedling stage. The phenotype of 17CL30 and 05S180 was investigated after 28 h of HS treatment. Figure 1A,B show that 17CL30 was more heat-tolerant than 05S180, and there was no evident difference between treatment group and control group of 17CL30. The leaves of 05S180 all wilted (Figure 1B), whereas a few leaves began to curl in 17CL30 (Figure 1A). The degree of cell membrane damage was measured according to the malondialdehyde (MDA) content. The heat treatment resulted in a significantly increased MDA content of both cultivars. However, the MDA content of heat-tolerant cultivar was still lower compared with the heat-sensitive cultivar (Figure 1C). The content of total soluble sugars, proline, total protein, and the main osmotic materials in plants were all increased after HS in both pepper cultivars. Furthermore, their contents in heat-sensitive genotype 05S180 were lower compared with the heat-tolerant genotype 17CL30 (Figure 1D–F).

### 2.2. Overview of Transcriptomic Data for 17CL30 and 05S180 

**Figure 2:**
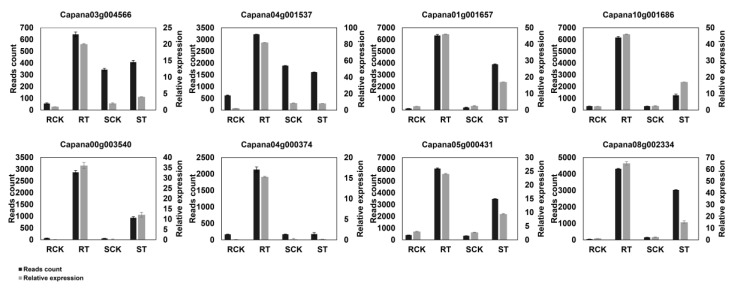
qRT-PCR analyses of eight genes. RCK refers to the control group of the 17CL30, RT refers to the heat treatment group of 17CL30, SCK refers to the control group of 05S180, ST refers to the heat treatment group of 05S180. *GST*: *Capana03g004566*, *NAC2*: *Capana04g001537*, *HSP20*: *Capana01g001657*, *HSP90*: *Capana10g001686*, *SLC*: *Capana00g003540*, *WRKY40*: *Capana04g000374*, *HSP20*: *Capana05g000431*, *GST*: *Capana08g002334*.

To investigate the transcriptome response to HS in pepper, the Illumina’s platforms were used to conduct high-throughput sequencing. By RNA-seq, 12 libraries were established (Supplementary Table S1). In short, a total of 334,699,604 and 342,395,306 clean reads were obtained from 17CL30 and 05S180, respectively (Supplementary Table S1). The Q20 of all libraries was >96.90%, the Q30 was >92.35, and the GC content was approximately 43% (Supplementary Table S1). To validate the RNA-seq data, eight genes were chosen for qRT-PCR, including *NAC2*, *HSP20*, *WRKY40*, *HSP90*, *HSP70*, *HSFA4*, and *GST*, which are related to HS response based on published studies. The results showed that the expression profiles of these genes were coincident with those of RNA-seq analysis (Figure 2).

### 2.3. Identification of Differentially Expressed Genes (DEGs) under HS

**Figure 3:**
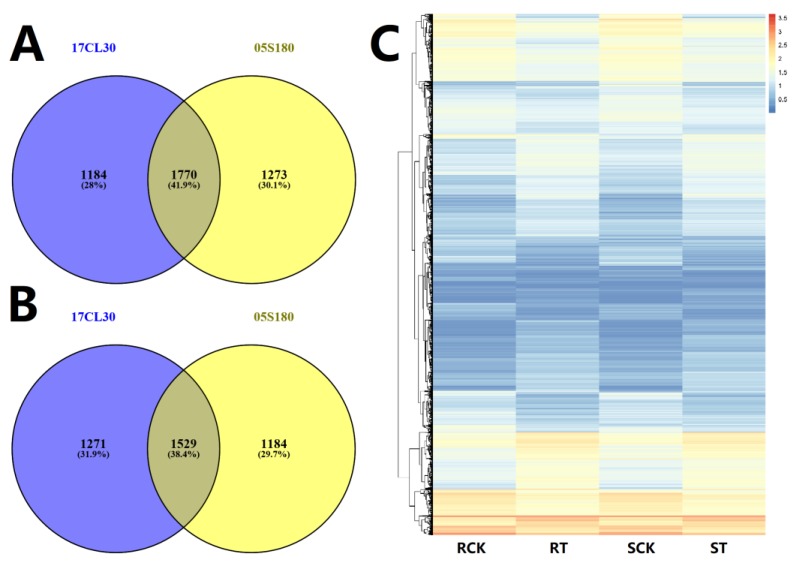
Analysis of transcriptome data. (**A**) Venn graph for 17CL30 (heat-tolerant cultivar) and 05S180 (heat-sensitive cultivar) of heat-induced genes. (**B**) Venn graph for 17CL30 and 05S180 of heat-repressed genes. (**C**) Heatmap of differentially expressed genes (DEGs) in RCK, RT, SCK, and ST. RCK refers to the control group of the 17CL30, RT refers to the heat treatment group of 17CL30, SCK refers to the control group of 05S180, ST refers to the heat treatment group of 05S180.

A total of 2954 up-regulated genes and 2800 down-regulated genes were identified in 17CL30 compared with the control group (Figure 3A,B). For 05S180 samples, there were 3043 up-regulated genes and 2713 down-regulated genes (Figure 3A,B). The gene expression pattern in 17CL30 and 05S180 was changed differently under HS. Meanwhile, there were some common DEGs in response to HS in two pepper cultivars, including 1770 (41.9%) heat-induced genes and 1529 (38.4%) heat-repressed genes (Figure 3A,B). To further investigate the transcriptomes of 17CL30 and 05S180 under HS, a heatmap for all DEGs under HS was generated (Figure 3C). The results showed that the gene expression patterns of 17CL30 and 05S180 were obviously changed when plants were exposed to HS (Figure 3C). 

### 2.4. The Common HS-Responsive Genes in 17CL30 and 05S180 

A total of 3299 DEGs were identified in both 17CL30 and 05S180 upon HS. We analyzed these 3299 DEGs to investigate the response mechanisms of pepper seedlings under HS. Some genes were more significantly induced in 17CL30 compared with 05S180 (Supplementary Table S2). Gene Ontology (GO) analysis showed that these DEGs could be significantly enriched (*q* value < 0.05) to seven, nine, and 14 terms from three branches, namely, molecular function, cellular component, and biological process, respectively (Supplementary Figure S1). The terms “3-oxo-arachidoyl-CoA synthase activity”, “3-oxo-cerotoyl-CoA synthase activity”, and “3-oxo-lignoceronyl-CoA synthase activity” were most highly enriched in the molecular function category, the terms “plastid chromosome”, “nucleoid”, and “plant-type cell wall” were mainly enriched in the cellular component category, while the terms “etioplast organization”, “transcription from plastid promoter”, and “very long-chain fatty acid metabolic process” were most enriched in the biological process category (Supplementary Figure S1).

**Figure 4:**
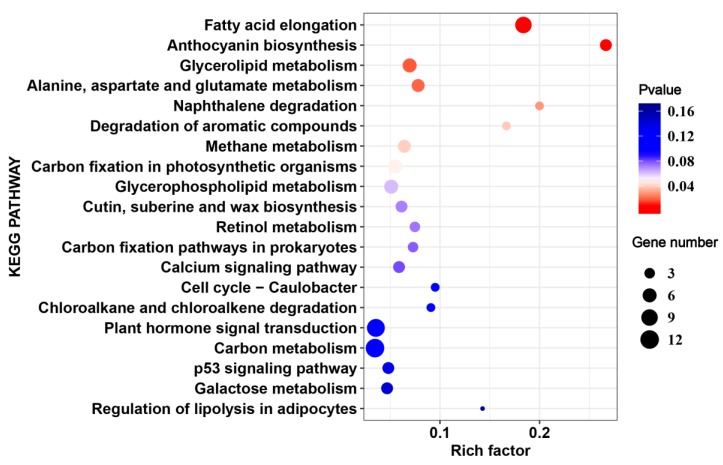
Top 20 enriched pathways for common heat-responsive genes in 17CL30 and 05S180.

Kyoto Encyclopedia of Genes and Genomes (KEGG) enrichment was carried out in order to better understand the biological functions of common HS-responsive genes. The KEGG analysis indicated that “fatty acid elongation”, “anthocyanin biosynthesis”, and “alanine, aspartate, and glutamate metabolism” were significantly enriched (*p* value < 0.05) by DEGs under HS (Figure 4, Supplementary Table S3). Moreover, “plant hormone signal transduction”, “cutin, suberine, and wax biosynthesis”, and “carbon fixation in photosynthetic organisms” were also enriched (Figure 4, Supplementary Table S3). 

### 2.5. Cultivar-Specific HS-Responsive Genes 

**Figure 5:**
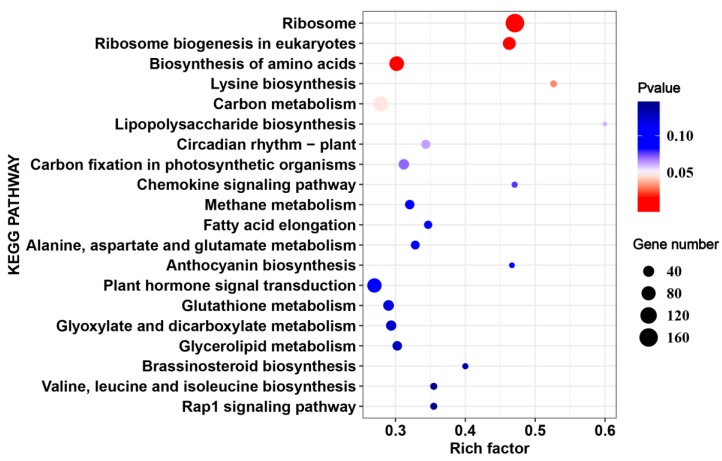
Top 20 enriched pathways for 17CL30-specific heat responsive genes.

**Figure 6:**
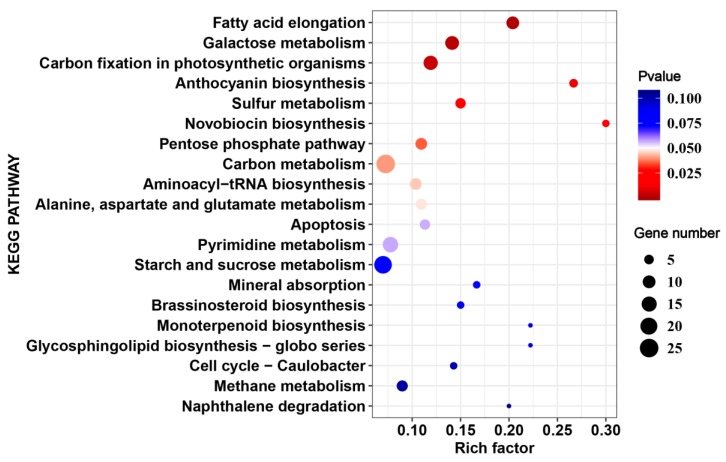
Top 20 enriched pathways for 05S180-specific heat responsive genes.

A total of 2455 DEGs were identified only from17CL30 under HS, including 1184 up-regulated and 1271 down-regulated genes (Figure 3A,B). However, 2457 DEGs were identified from 05S180, including 1273 up-regulated and 1184 down-regulated genes (Figure 3A,B). We also explored the possible mechanism underlying the different phenotypes in 17CL30 and 05S180 when seedlings were subjected to HS. KEGG enrichment of specific DEGs clearly indicated that 17CL30-specific DEGs were significantly enriched in “ribosome”, “ribosome biogenesis in eukaryotes”, “biosynthesis of amino acids”, and “lysine biosynthesis”, while 05S180-specific DEGs were enriched in “fatty acid elongation”, “galactose metabolism”, and “carbon fixation in photosynthetic organisms” (Figure 5 and Figure 6, Supplementary Table S4). 

### 2.6. Analysis of HS-Responsive TFs 

TFs play an important role in plants in response to HS by regulating the expressions of target genes. In order to identify the TFs involved in HS response, we analyzed the TFs of DEGs identified from both cultivars. A total of 425 TFs were differently expressed under HS in 17CL30 and 05S180 (Supplementary Table S5), indicating the key roles of TFs related to HS response. These 425 TFs were divided into 29 families. The major TFs identified in this study included *MYB* (45), *AP2/ERF* (44), *C2H2* (30), *bHLH* (28), *WRKY* (20), *NAC* (18), *bZIP* (14), *TCP* (14), and *HSF* (10). Among these families, two *TCPs* were up-regulated, while 12 *TCPs* were down-regulated. In addition, eight *HSFs* were up-regulated, while two *HSFs* were down-regulated. Furthermore, most TFs were down-regulated in both cultivars to different degrees. For example, 17 *AP2s* were up-regulated, 27 *AP2s* were down-regulated, 13 *MYBs* were up-regulated, and 32 *MYBs* were down-regulated. 

### 2.7. Metabolomic Analsis during HS in 17CL30 and 05S180

**Figure 7:**
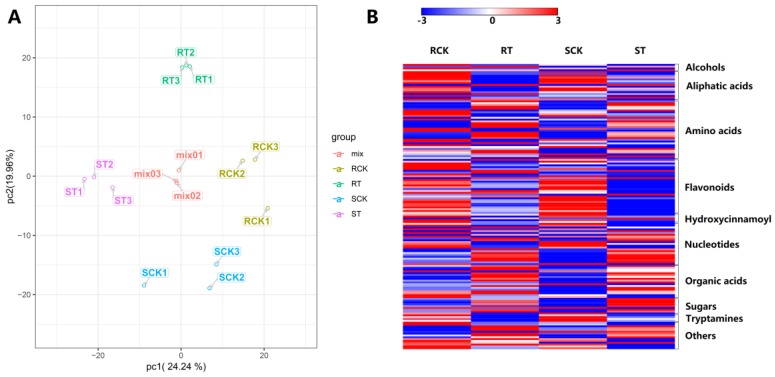
(**A**) Metabolic analysis using a principal component analysis (PCA). (**B**) Heat map of all differentially accumulated metabolites (DAMs) for RCK, RT, SCK, ST. RCK refers to the control group of the 17CL30, RT refers to the heat treatment group of 17CL30, SCK refers to the control group of 05S180, ST refers to the heat treatment group of 05S180, mix refers to the quality control (QC) samples that were mixed with equivalent test samples, 1, 2, and 3 refer to the three replicates.

Metabolite reprogramming caused the phenotypic changes directly. In order to explore the variation of metabolomic profile in response to HS and understand the different heat-tolerant phenotypes, we analyzed metabolites of 17CL30 and 05S180 using LC-MS/MS. A total of 717 metabolites were identified, which could be classified into 32 categories (Supplementary Table S6). Among these metabolites, “organic acids”, “amino acid derivatives”, “nucleotide and its derivates”, and “flavone” were the top four accumulated metabolites (Supplementary Table S6). In a principal component analysis (PCA) model based on three quality control (QC) samples (mix) and 12 test samples, the first two principal components could separate 15 samples clearly, accounting for 52.6% of the total variability (Figure 7A). The PCA1 accounted for 23.86% of the variability, whereas the PCA2 accounted for 28.74% of the variability (Figure 7A). We also generated a heatmap of all metabolites to show the changes of metabolites in 17CL30 and 05S180 under HS (Figure 7B). The results clearly showed that the changes of metabolites in two materials were significantly different, indicating that the response to HS was obviously also different.

**Figure 8:**
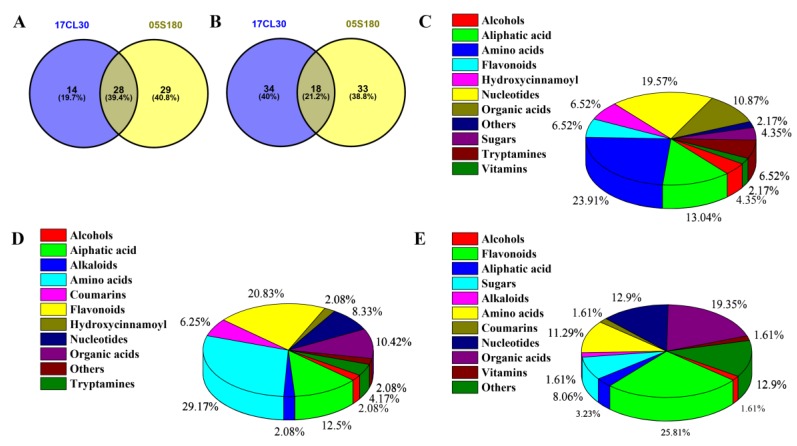
Classification of identified DAMs. (**A**) Venn for up-regulated metabolites between 17CL30 and 05S180. (**B**) Venn for down-regulated metabolites between 17CL30 and 05S180. (**C**) Pie graph for shared metabolites between 17CL30 and 05S180. (**D**) Pie graph for specific metabolites in 2144. (**E**) Pie graph for specific metabolites in 05S180.

Compared with the control group, 94 metabolites (42 up-regulated and 52 down-regulated ones) were identified as differentially accumulated metabolites (DAMs) in 17CL30 under HS (Figure 8A, Supplementary Table S7). In contrast, 108 metabolites (57 up-regulated and 51 down-regulated ones) were identified as DAMs in 05S180 (Figure 8B, Supplementary Table S7). Among these metabolites, 46 metabolites were identified in both cultivars. Conversely, 48 metabolites were identified only in 17CL30, and such a number became 62 in 05S180 (Figure 8A,B). By checking the DAMs, several amino acids shared by 17CL30 and 05S180 were increased during HS (Figure 8C). Several of them in 17CL30, such as citrulline, serine, cysteine, and glutamine, were more significantly induced compared with those in 05S180. However, there was no significant difference in the increase in some amino acids in both cultivars, including homocitrulline, alanine, and ornithine. The 17CL30-specific metabolomes mainly included amino acids (29.17%), flavones (20.83%), and aliphatic acid (12.5%). In contrast, the 05S180- specific metabolomes mainly included flavones (25.81%), organic acids (19.35%), and amino acids (11.29%) (Figure 8D,E). 

### 2.8. Integrated Analysis between DEGs and DAMs

**Figure 9:**
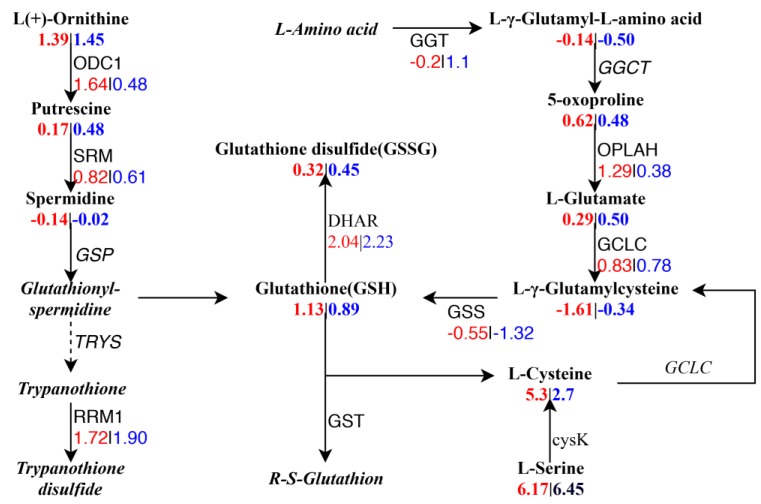
Network of the glutathione metabolism pathway. The location of the node is metabolites and the substances on the line are genes. Metabolites and genes in italics were not detected. Some of them that were unaffected by HS have been omitted from this graph. The red numbers represent the |log2fold change| of genes or metabolites for control and HS in 17CL30, while the blue number represents the |log2fold change| of genes or metabolites for control and HS in 05S180.

Supplementary Table S8 shows the correlation between genes and metabolites based on integrated analysis. In order to better understand the relationship between genes and metabolites, DEGs (Figure 3A,B) and DAMs (Figure 8A,B) that belong to the same group, were mapped to the KEGG pathway map (Supplementary Table S9). There were 130 and 97 pathways enriched in 17CL30 and 05S180, respectively. Interestingly, we found that “glutathione metabolism” was markedly affected and the detailed network of this pathway was mapped (Figure 9). In this pathway, we identified eight genes and 11 metabolites. Glutathione (GSH) content was dramatically increased under HS, whereas there was no significant change in the content of GSH in 05S180. In addition, *GSS* (*Capana08g001413*) and *DHAR* (*Capana05g002401*), key genes in this pathway, were identified. GSS was down-regulated, while DHAR was up-regulated after 28 h of HS. Glutathione S-transferases (GSTs) were also induced by HS. In our study, the expressions of *GSTs* (*Capana03g004566*, *Capana09g001861*, and *Capana00g002164*) were higher in 17CL30 than those in 05S180 under HS. 

## 3. Discussion

Temperature is one of the most important impact factors restricting agriculture development. Crop failures occur worldwide in recent years due to the elevated temperature. When plants are subjected to HS, more osmotic adjustment substances, such as inorganic ion, soluble sugars, proline, and betaine, are accumulated to reduce heat-induced damage [44]. Our current study was designed to explore the response pattern of pepper to HS and the possible mechanism for the different heat-resistance in 17CL30 and 05S180. Under HS of 40 °C, both pepper varieties showed different phenotypes, and 17CL30 was more heat tolerant compared with 05S180 (Figure 1A). We determined the contents of the total soluble sugars, MDA, proline and total protein of leaves after 28 h of HS. The MDA content was increased in both pepper cultivars, while its content in the heat-tolerant cultivar (17CL30) was lower than that of the heat-sensitive cultivar (05S180) (Figure 1B). HS enhanced the peroxidative degree of membrane lipid, evidenced by increased MDA content [45,46]. Moreover, the heat-sensitive genotypes suffered much more from membrane injury than heat-tolerant genotypes [9]. The main osmotic adjusting materials in plants (total soluble sugars, proline, and total protein) were all increased after HS in both pepper cultivars. However, their contents in heat-sensitive cultivar 05S180 were lower than those of the heat-tolerant cultivar 17CL30. Numerous studies have indicated that osmotic adjusting materials play key roles in plant response to HS. According to the changes in these four physiological indices, we concluded that 17CL30 was more heat tolerant. Moreover, we obtained accurate data from transcriptomic and metabolomic analyses based on RNA-seq and LC-MS/MS, respectively. We analyzed the genes and metabolites of metabolic pathways, which were significantly affected by HS.

A set of studies has demonstrated that HS signaling is transduced through multiple signaling pathways to activate TFs, and then a lot of *HSPs* and other HS-responsive genes are induced to cope with the HS [47,48]. Based on the published studies, *HSFA4* represents an activator of HS-responsive genes [49]. In our study, *HSFA4* (*Capana07g001899*) was up-regulated in 17CL30, while this gene was down-regulated in 05S180. Previous studies have shown that HS promote the expressions of *HSP20*, *HSP70* and *HSP90* in *Brassica rapa* [50], pepper [9], and wheat [10]. HSPs play a vital role in the protection of cell metabolic apparatus and they also function as key factors for plants in response to HS [28]. In the present study, *HSPs* were remarkably induced by HS. A total of 29 *HSPs* (18 *HSP20*, eight *HSP70,* and three *HSP90*) were identified as DEGs in 17CL30 after HS, whereas 26 *HSPs* (17 *HSP20*, seven *HSP70,* and two *HSP90*) were differently expressed in 05S180. Interestingly, the expressions of these genes in 17CL30 were significantly higher than those in 05S180. These differences might reasonably explain why 17CL30 was much more heat tolerant than 05S180. Meanwhile, some members of the *AP2/ERF*, *bHLH*, *MYB*, *WRKY*, *NAC,* and *bZIP* genes were up-regulated in 17CL30 compared with 05S180 after HS, showing that these TFs might play an important role in HS response. *WRKY40* is a positive regulator of HS, and the over-expression of *CaWRKY40* enhances resistance to HS in tobacco [51]. In addition, *WRKY6* binds to and activates the *WRKY40* promoter to regulate HS tolerance in pepper [52]. In our study, *WRKY40* (*Capana12g001134*) was significantly induced by HS in 17CL30, suggesting that 17CL30 was more heat tolerant than 05S180. *WRKY6* (*Capana02g002230*) was up-regulated in 17CL30, while it was down-regulated in 05S180. Furthermore, *NACs* (*Capana04g001537* and *Capana05g000569*) were also induced in heat-tolerant cultivar 17CL30, while they were suppressed in heat-sensitive cultivar 05S180. Consistent with our findings, *NAC* is induced by HS, and the over-expression of *NAC* in rice results in increased tolerance to HS [26]. Collectively, the expressions of genes were typically much more significantly changed in 17CL30 compared with 05S180, which might help explain why 17CL30 was more heat tolerant than 05S180. 

Metabolites are the final products of cell activities, which directly reflect the impact of environmental changes or physiological and pathological changes on plants [37]. The liquid chromatography-electrospray ionization-tandem mass spectrometry system (LC-ESI-MS/MS) was employed for qualitative and quantitative analysis of widely targeted metabolites in dried pepper leaf samples affected by HS. In our study, some soluble sugars, such as D-glucoronic acid, were more significantly up-regulated in 17CL30 compared with 05S180 under HS. On the contrary, some soluble sugars were down-regulated. These findings could be attributed to the fact that peppers still need to consume sugars to maintain growth under short-term HS [53]. Both 05S180 and 17CL30 showed no significant differences in the accumulation of some amino acids, including citrulline, homocitrulline, and ornithine, whereas cysteine and glutamine were accumulated to a much greater extent in 17CL30. This finding was consistent with previous research that soluble sugars and some amino acids are decreased under HS [37,54]. Flavonoids also play an important role in alleviating HS response and are considered as antioxidants to eliminate reactive oxygen species (ROS) produced under HS [55,56]. In our study, flavonoids (isorhamnetin-3-O-neohesperidoside, daidzein, 7-O-methyleriodicty-ol, and tulipanin) were synthesized to reduce the heat-induced damage in both 17CL30 and 05S180. These results were consistent with previous studies that an increasing trend of flavonoid synthesis is found in pepper under HS [56]. The same conclusions have been also obtained from the studies on carrot [57] and *Pinus radiata* [53] in response to HS. Ureido-isobutyric acid may play a key role in HS response and is the most accumulated organic acid. Organic acids are considered as a class of substances, which are involved in HS tolerance [58]. Therefore, it is necessary to explore the specific function of ureido-isobutyric acid in further research.

By integrating the transcriptomic data with metabolomic data, we obtained a great deal of information about the metabolic pathway (KEGG). The GSH metabolic pathway was one of the most enriched metabolic pathways after HS. GSH, which is composed of glutamate, cysteine, and glycine, plays a central role in maintaining the dynamic balance between oxidation and antioxidation in plants subjected to stress-induced oxidative injury [59,60]. Our results suggested that the cysteine content was increased under HS and accumulated to 38-fold in 17CL30 under HS, while an increase of only 6-fold was detected in 05S180. Cysteine is synthesized from serine by the key enzyme cysteine synthase A (cysK). Our study also showed that pepper regulated cysteine accumulation during HS by inducing the genes encoding cysK. Three related genes (*Capana03g003700*, *Capana08g001327,* and *Capana00g001461*) were all up-regulated by 3~4-fold under HS in 17CL30 compared with the control group, whereas only one gene (*Capana00g001461*) was increased by 1-fold under HS in 05S180. Meanwhile, the GSH content was also more significantly increased and accumulated in 17CL30. It is well known that GSTs can reduce the damage of toxic substances caused by various forms of stress by catalyzing the binding of GSH and hydroxyl radical, and the oxidation products of membrane lipids and other metabolites [61,62]. The up-regulation of *GSTs* occurred after HS, and the expressions of *GSTs* in 17CL30 were much higher compared with 05S080. As a result, 17CL30 was much more heat tolerant than 05S180. Studies on wheat heat adaptation have also shown that GSTs are significantly accumulated in response to HS [63]. These findings are also consistent with the results of Labrou [64] and Lee [65]. 

In the present study, transcriptomic variation and metabolomic reprogramming revealed the complex response mechanisms induced by HS. Moreover, we also elucidated the GSH metabolic pathway related to the tolerance of pepper under HS. These results provide valuable insights into the HS response mechanisms of pepper and other Solanaceae crops. 

## 4. Materials and Methods 

### 4.1. Plant Materials and Heat Treatments

Two pepper cultivars, heat-tolerant 17CL30 and heat-sensitive 05S180, were obtained from Vegetable Institution of Hunan Academy of Agricultural Science. Seeds were sown in 10 cm × 10 cm plastic pots with the nutrient substrate. Plants were grown in a light incubator under optimum growth conditions (~28 ℃ with 16 h light and ~20 ℃ with 8 h dark) until the plants grew to the six-leaf stage. For the heat treatment, plants were cultivated at ~40 ℃ for 28 h. Such a treatment duration was chosen because the phenotypic differences between the two cultivars were greatest at that duration (Figure 1A,B). Control groups were grown in the incubator under the same conditions. The top three-leaf samples were harvested (three replicates) after heat treatment, immediately frozen in the liquid nitrogen and stored at −80 ℃ prior to RNA-Seq and metabolite extraction. Each sample was composed of leaves from 20 plants. Meanwhile, the samples were named as RCK (control group of the 17CL30), RT (heat treatment group of the 17CL30), SCK (control group of the 05S180), and ST (heat treatment group of the 05S180).

### 4.2. mRNA-seq Library Constuction and RNA Sequencing

Total RNA was extracted from 12 pepper leaf samples using TRIzol reagent (Invitrogen, Carlsbad, CA, USA) according to the manufacturer’s instructions. mRNA with poly (A) was isolated by using oligo (dT) beads and randomly interrupted into short fragments. The first-strand cDNA was synthesized by the M-MuLV reverse transcriptase system using these RNA fragments as templates and random hexamer primers. dNTPs were used as raw materials to synthesize the second-strand cDNA using DNA polymerase I and RNaseH. The double-strand cDNA fragments were purified and then connected with sequencing adapters. The fragment of 300 bp was selected using AMPure XP beads. Subsequently, the cDNA libraries were constructed after PCR amplification. The quality and quantity of the cDNA libraries for sequencing were tested using the Agilent 2100 bioanalyzer system (Agilent Technologies, Palo Alto, CA, USA). Qualified libraries were sequenced with the Illumina HiSeq 4000 platform (Illumina, Foster, CA, USA).

### 4.3. Read Alignment and Analysis 

To efficiently and accurately analyze the sequencing results, the raw reads were filtered by deleting low-quality reads (the reads with ambiguous nucleotides > 10%, and reads in which the low quality (Q ≤ 5) base number > 50%), adapter sequences and sequences with more than 10% poly-N using fastp program [66] with the default parameters. Raw reads from every library were mapped to the pepper reference genome (Zunla-1 version 2) [6] using HISAT2 [67]. Raw counts of genes were performed using featureCounts [68]. Genes with a |log2fold change| ≥ 1 and false discovery rate (FDR) < 0.05 were identified as DEGs by using DESeq2 [69]. Finally, the functional annotation of DEGs was carried out with the GO (http://geneontology.org/) and KEGG databases (https://www.genome.jp/kegg). The GO analysis of DEGs was performed via R package clusterProfiler [70]. The KEGG pathway analysis was performed by BLAST software [71], and the enrichment analysis of the KEGG pathway was carried out by KOBAS 2.0 software [72] based on hypergeometric distribution. iTAK software [73], which integrated PlnTFDB [74] and PlantTFDB [75] databases, was used to predict the TFs among DEGs in this study. 

### 4.4. Sample Preparation and Metabolite Detection

The pepper leaf samples were crushed into the powder after vacuum freeze-drying using a mixer mill MM400(Retsch Technology, Haan, Germany) with zirconia beads (15 mm) for 1.5 min at 30 Hz. Subsequently, 100 mg leaf powder was extracted overnight at 4 ℃ in 1.0 mL 70% aqueous methanol. Next, samples were centrifuged at 10,000 g for 10 min, and the extractives were absorbed, filtered and transferred to a new tube for LC-MS analysis. The QC samples were mixed by all test samples and inserted into each test sample to check the repeatability of the analytical process.

The LC-ESI-MS/MS system was used to analyze the extractives, which was composed of HPLC (Shim-pack UFLC SHIMADZU CBM30A system, http://www.shimadzu.com.cn/) and MS (Applied Biosystems 6500 Q TRAP, http://www.appliedbiosystems.com.cn/). The experimental conditions were as follows: chromatographic column, Waters ACQUITY UPLC HSS T3 C18 (1.8 µm, 2.1 mm*100 mm), solvent system, water with 0.04% acetic acid (A), acetonitrile with 0.04% acetic acid (B), gradient program, 95:5 *V/V* at 0 min, 5:95 *V/V* at 11.0 min and kept for 1 min, 95:5 *V/V* at 12.1 min and kept for 3 min, constant flow rate, 0.4 mL/min, column temperature, 40 °C, injection volume: 2 μL. 

The effluent was connected to electrospray ionization (ESI)-triple quadrupole-linear ion trap (QTRAP) MS/MS (ESI-Q TRAP-MS/MS), alternatively. Linear ion trap (LIT) and triple quadrupole (QQQ) scans were acquired on a triple Q TRAP. Mass spectrometry conditions were set as follows: the ESI temperature was set as 500 °C, the ion spray voltage was 5500 V, and ion source gas I (GSI), gas II (GSII) and curtain gas (CUR) were set as 55, 60, and 25.0 psi, respectively. The collision gas was set at high. In addition, 10 and 100 μmol/L polypropylene glycol solutions were used for instrument tuning and mass calibration was performed in QQQ and LIT modes, respectively. QQQ scans were acquired as multi-reaction monitoring (MRM) experiments with collision gas (nitrogen) of 5 psi. In QQQ, the declustering potential (DP) and collision energy (CE) of individual MRM transitions were tested with further DP and CE optimization. The obtained data were processed by mass spectrometry software Analyst 1.6.1 (Applied Biosystems Company, Framingham, MA, USA) 

### 4.5. Metabolite Profiling

Metabolite profiling was carried out using a widely targeted metabolomic method based on the self-built database MWDB (Metware biotechnology Co., Ltd. Wuhan, China) (http://www.metware.cn/). This method has been described in previous studies [76,77]. The metabolites were qualitatively analyzed according to the secondary spectrum information. Moreover, metabolite quantification was accomplished by MRM mode analysis using triple quadruple-bar mass spectrometry. PCA was used to analyze the variability between groups and within groups. Partial least squares-discriminant analysis (PLS-DA) was performed to DAMs. Metabolites satisfying |log2fold change| ≥1 and variable importance of the projection (VIP) ≥1 were defined as DAMs. The functional annotation of DAMs was performed based on KEGG.

### 4.6. Measurements of Total Soluble Sugar, MDA, Proline and Total Protein

After 28 h of treatment, samples were harvested, and the contents of total soluble sugar, MDA, free proline and total protein in the leaves of 17CL30 and 05S180 were determined. The content of total soluble sugar was determined using the anthrone method [33]. MDA content was measured to evaluate the peroxidation of membrane lipids using a previously established method [31]. The content of proline was analyzed using a previously established method [31]. The content of total protein was assayed as previously described [78]. 

### 4.7. Integrated Analysis between HS-Responsive Genes and Metabolites

Transcriptomic and metabolomic data were uniformly normalized by log2 transformation [77]. The Pearson correlation analysis between DEGs and DAMs was evaluated based on the cor function of R language with normalized data. The KEGG enrichment analysis was carried out using DEGs with DAMs |Pearson Correlation Coefficient (PCC| > = 0.8). Then DEGs and DAMs, which were divided into the same group, were mapped to the KEGG pathway map. 

### 4.8. Synthesis of cDNA and qRT-PCR

Briefly, cDNA was synthesized with 2 µg RNA using the one-step transcription Kit (Vazyme, Nanjing, China) according to the manufacturer’s instructions. To verify the results of RNA-Seq, eight DEGs were randomly selected, including genes related to HS response. The primers for qRT-PCR are listed in Supplementary Table S10. The qRT-PCR was carried out using a LightCycler ^®^ 96 Real-Time PCR System (Roche, Basel, Switzerland), and the relative expression levels of target genes were calculated using the 2^−ΔΔCt^ method. Pepper gene *β-Actin* was selected as the housekeeping gene. 

## 5. Conclusions

Cultivars 17CL30 and 05S180 are two closely related pepper varieties, but their heat resistance is quite different. Briefly, the multi-omics analysis provided a great deal of information for new metabolites and genes related to HS. As a result, we found that TFs were changed during HS, which could be responsible for activating the HS-responsive mechanism through a complicated regulation network. Importantly, both transcriptomic and metabolomic data indicate that the higher accumulation ability of common genes and metabolites might be one of the primary reasons contributing to heat resistance in 17CL30. Moreover, it is possible that there might be some heat regulatory pathways in 17CL30 that do not exist in 05S180. Our results help us better understand the extremely complex regulatory mechanisms in the process of HS responses in plants.

## Supplementary Materials

Supplementary materials can be found at https://www.mdpi.com/1422-0067/20/20/5042/s1.

## Abbreviations

HS	Heat stress
DEGs	Differentially expressed genes
DAMs	Differentially accumulated metabolites
HSPs	Heat shock proteins
HSFs	Heat shock transcription factors
sHSPs	Small HSPs
TFs	Transcription factors
MDA	Malondialdehyde
RCK	Control group of 17CL30
RT	Heat treatment group of 17CL30
SCK	Control group of 17CL30
ST	Heat treatment group of 05S180
GO	Gene Ontology
KEGG	Kyoto Encyclopedia of Genes and Genomes
PCA	Principal component analysis
QC	Quality control
GSH	Glutathione
FDR	False Discovery Rate
ESI	Electrospray ionization
QTRAP	Quadrupole-linear ion trap
LIT	Linear ion trap
QQQ	Triple quadrupole
MRM	Multi-reaction monitoring
DP	Declustering potential
CE	Collision energy
PLS-DA	Partial least squares-discriminant analysis
VIP	Variable importance of the projection
PCC	Pearson Correlation Coefficient
